# Evaluation of different nested PCRs for detection of *Anaplasma phagocytophilum* in ruminants and ticks

**DOI:** 10.1186/s12917-016-0663-2

**Published:** 2016-02-24

**Authors:** Jifei Yang, Zhijie Liu, Qingli Niu, Junlong Liu, Jingying Xie, Qiuyu Chen, Ze Chen, Guiquan Guan, Guangyuan Liu, Jianxun Luo, Hong Yin

**Affiliations:** State Key Laboratory of Veterinary Etiological Biology, Key Laboratory of Veterinary Parasitology of Gansu Province, Lanzhou Veterinary Research Institute, Chinese Academy of Agricultural Science, Xujiaping 1, Lanzhou, Gansu 730046 P. R. China; Jiangsu Co-innovation Center for Prevention and Control of Important Animal Infectious Diseases and Zoonoses, Yangzhou, 225009 P. R. China

**Keywords:** *A. phagocytophilum*, Diagnosis, PCR, Ruminants, Tick, Prevalence

## Abstract

**Background:**

*Anaplasma phagocytophilum* is a causative agent of granulocytic anaplasmosis in mammals, which has a broad geographical distribution and a high degree of clinical diversity. Currently, numerous PCR assays have been developed and used for the detection of *A. phagocytophilum* in various specimens. However, their performance varies. The aim of this study was to evaluate the performance of five nested PCR assays by detection of 363 ruminant and tick samples, and to select the most appropriate methods for the sensitive detection of *A. phagocytophilum* in environmental or clinical samples.

**Results:**

Positive PCR results for *A. phagocytophilum* were obtained in 75 (20.7 %), 42 (11.6 %) and 19 (5.2 %) specimens with primer sets EC (EC9/EC12a and SSAP2f/SSAP2r), EE (EE1/EE2 and EE3/EE4) and ge (ge3a/ge10r, ge9f/ge2), respectively. The amplification of template DNA with the primer set MSP (MAP4AP5/MSP4AP3, msp4f/msp4r) could not be obtained in both ruminants and ticks, and a low specificity of the EL primers [EL(569)F/EL(1193)R, EL(569)F/EL(1142)R] in tick samples was observed. Our results revealed that the nested PCR with primer set EC complementary to the *16S rRNA* gene was the most sensitive assay for detection of *A. phagocytophilum* in ruminant and tick specimens. *A. phagocytophilum* was detected in 47 (35.1 %) sheep, 12 (10.4 %) cattle, and 17 (14.9 %) ticks. Two *A. phagocytophilum* genotypes were identified, that varied between sheep and cattle in sample collection sites.

**Conclusions:**

This report provides more valuable information for the diagnosis and management of granulocytic anaplasmosis in China.

**Electronic supplementary material:**

The online version of this article (doi:10.1186/s12917-016-0663-2) contains supplementary material, which is available to authorized users.

## Background

HGA (Human granulocytic anaplasmosis) is an emerging tick-borne zoonosis caused by the obligate intracellular bacterium *Anaplasma phagocytophilum* (formerly known as *Ehrlichia phagocytophila*, *Ehrlichia equi* or the HGE agent) [1, 2]. The organism is commonly maintained in nature through an enzootic cycle involving ticks and vertebrate hosts [3, 4]. Several *Ixodes* ticks are known or suspected vectors of *A. phagocytophilum*, including *Ixodes scapularis* and *Ixodes pacificus* in North America, *Ixodes ricinus* in Europe, and *Ixodes persulcatus* in Russia and Asian [3, 5–8]. *A. phagocytophilum* infects a variety of hosts and causes granulocytic anaplasmosis in humans but also in wild and domestic animals [9, 10]. Since the first suspected human case was reported in Anhui province in 2006, more than 90 cases of HGA have been recorded in Beijing, Tianjin, Shandong, Henan, Hubei and Inner Mongolia in China [11, 12]. The actual number of human cases may be much higher due to the poor diagnostic tools, non-specificity of the reported symptoms and lack of awareness of public health professionals [13].

Rapid and sensitive detection of *A. phagocytophilum* is an essential step for the control and prevention of HGA in endemic areas. *A. phagocytophilum* was initially identified as a human agent using molecular methods rather than culture or serological tests [1, 14]. Since then, PCR assays have played an important role in the laboratory diagnosis of HGA in clinical and environmental specimens for their rapidity and relative ease of performance. However, their performance varies significantly [15, 16]. Most studies have always focused on the analytical sensitivity or specificity of those PCR assays, and the ability to detect small amounts of nucleic acid and specific nucleic acid fragments, to enable distinction of closely related strains [15, 16]. However, considerable variation within *A. phagocytophilum* strains has been described, and isolates from various hosts or geographic locations have displayed genetic diversity and divergence within frequently used PCR-target genes, such as *groESL*, *ankA* and *msp4* [17–19]. Some assays could not detect all the variants or ecotypes of *A. phagocitophilum*. Thus, it is critical to evaluate the performance of prospective assays in certain species and in a given geographic area in order to obtain more reliable results. The objective of this study was to evaluate five nested PCRs for detection of *A. phagocytophilum* in ruminants and tick specimens from northwest China.

## Methods

EDTA–K^2+^ anticoagulated blood samples were taken from the jugular vein of 249 asymptomatic domestic ruminants (134 sheep and 115 cattle) and collected in a sterile tube in May 2015 from Ili Kazakh Autonomous Prefecture, in northern Xinjiang, China. One hundred and fourteen ticks were collected from sheep, cattle and other livestock within same herds. Five species of ticks were identified in accordance with the standard taxonomic keys [20]. Seventy-two ticks collected from cattle were identified as *Dermacentor marginatus*; 35 ticks collected from sheep were identified as *Haemaphysalis punctata* (*n* = 28), *Haemaphysalis concinna* (*n* = 3) and *Hyalomma asiaticum* (*n* = 4); and seven ticks collected from horses were identified as *Hyalomma detritum*. The samples collection in the present study was consented by animals owners. All animal treatments and handling complied with Ethical Guidelines and were approved by the Animal Ethics Committee of Lanzhou Veterinary Research Institute, Chinese Academy of Agricultural Sciences.

DNA was extracted from blood and tick samples using a Gentra Puregene DNA Purification kit (Qiagen) as described previously [9]. All DNA samples were examined for the presence of *A. phagocytophilum* by nested PCRs. The PCR primers are listed in Table 1. The reaction was performed in an automatic thermocycler (Bio-Rad, Hercules, CA, USA) in a total volume of 25 μL containing 2.5 μL of 10× PCR buffer (Mg^2+^ Plus), 2.0 μL of each dNTP at 2.5 mM, 1.25 U of *Taq* DNA polymerase (TaKaRa, Dalian, China), 2.0 μL of template DNA, 1.0 μL of each primer (20 pmol) and 16.25 μL of distilled water. DNA from sheep infected with *A. phagocytophilum* validated by sequencing (Gene accession no. JN558811) was used as a positive control, and sterile water was used as the blank control for each run. The cycling conditions for the first and second round amplification involved 4 min of denaturation at 94 °C, 35 cycles at 94 °C for 30 s, annealing for 30 s at a temperature dependent on the primers applied (annealing temperatures shown in Table 1), and 72 °C for 1 to 1.5 min (dependent on the length of target fragments), with a final extension step at 72 °C for 10 min. The PCR products were visualized by UV transillumination in a 1.5 % agarose gel following electrophoresis and staining with ethidium bromide.

**Table 1:** Oligonucleotide primers used for detection of *A. phagocutophilum*

Target gene	Primer name	Primer Sequence (5’-3’)	Annealing temp (°C)	Amplicon size (bp)	Reference
*16S rRNA*	ge3a	CACATGCAAGTCGAACGGATTATTC	55	932	[25]
	ge10r	TTCCGTTAAGAAGGATCTAATCTCC			
	ge9f	AACGGATTATTCTTTATAGCTTGCT	55	546	
	ge2	GGCAGTATTAAAAGCAGCTCCAGG			
*Msp4*	MAP4AP5	ATGAATTACAGAGAATTGCTTGTAGG	54	849	[24, 27]
	MSP4AP3	TTAATTGAAAGCAAATCTTGCTCCTATG			
	msp4f	CTATTGGYGGNGCYAGAGT	55	381	
	msp4r	GTTCATCGAAAATTCCGTGGTA			
*16S rRNA*	EE-1	TCCTGGCTCAGAACGAACGCTGGCGGC	50	1433	[23]
	EE-2	AGTCACTGACCCAACCTTAAATGGCTG			
	EE-3	GTCGAACGGATTATTCTTTATAGCTTGC	50	926	
	EE-4	CCCTTCCGTTAAGAAGGATCTAATCTCC			
*groEL*	EL(569)F	ATGGTATGCAGTTTGATCGC	62	624	[26, 34]
	EL(1193)R	TCTACTCTGTCTTTGCGTTC			
	EL(569)F	ATGGTATGCAGTTTGATCGC	56	573	
	EL(1142)R	TTGAGTACAGCAACACCACCGGAA			
1*6S rRNA*	EC9	TACCTTGTTACGACTT	55	1462	[35]
	EC12a	TGATCCTGGCTCAGAACGAACG			
	SSAP2f	GCTGAATGTGGGGATAATTTAT	55	641	
	SSAP2r	ATGGCTGCTTCCTTTCGGTTA			

*A. phagocytophilum* positive samples were selected randomly and verified by sequencing. The PCR products were cloned into the pGEM-T Easy vector (Promega, Shanghai, China) and subjected to bidirectional sequencing (Sangon Biotech, Shanghai, China). The sequences obtained were compared with reference sequences from GenBank (see Additional file 1). A phylogenetic tree was then constructed using the neighbor-joining (NJ) algorithm with the Kimura two-parameter model of the Mega 4.0 Software [21]. The GenBank accession numbers for the partial 16S rRNA gene sequences obtained in this study were as follows: KT944028-KT944029 and KT951192.

The results were analyzed using a Chi-square test in Predictive for Analytics Software (PASW) Statistics version 18. A difference was considered statistically significant at *P* < 0.05.

## Results

Of the total 363 ruminant and tick specimens that were included in our evaluation of the five nested PCR assays, positive PCR results for *A. phagocytophilum* were obtained in 75 (20.7 %), 42 (11.6 %) and 19 (5.2 %) specimens with primer sets EC (EC9/EC12a and SSAP2f/SSAP2r), EE (EE1/EE2 and EE3/EE4) and ge (ge3a/ge10r, ge9f/ge2), respectively (Table 2). DNA of *A. phagocytophilum* was found in only two cattle specimens using primer set EL [EL(569)F/EL(1193)R, EL(569)F/EL(1142)R]. However, unspecific products were obtained in tick specimens with EL primers as a result of two bands between 250 and 500 bp (data not shown). Under the PCR conditions outlined, amplification of the template DNA with the primer set MSP (MAP4AP5/MSP4AP3, msp4f/msp4r) could not be obtained (Table 2). Apart from the primer sets EL and MSP, the assays showed that the positive rates of *A. phagocytophilum* infection in ruminant and tick specimens ranged from 5.6 to 20.7 %. The highest positive rate (20.7 %, 75/363) was observed using the EC primer set (Table 2). The PCR with EC primer set was more sensitive than the ones with EE and ge primer sets (Chi2 = 39.944, df = 2, *P* < 0.001); and the PCR with EE primer set was more sensitive than that with ge primer set (Chi2 = 9.468, df = 1, *P* < 0.01). Moreover, an additional tick sample (2–21) was negative with EC primers but positive with EE, which gave an overall positivity rate of 20.9 % in our sample population. As shown in Table 2, the infection rates of *A. phagocytophilum* were 35.1 %, 10.4 % and 14.9 % in sheep, cattle and ticks, respectively. Of those tick samples, *A. phagocytophilum* was detected in *D. marginatus* collected from cattle and *H. punctata* collected from sheep.

**Table 2:** *A. phagocytophilum* in ruminants and ticks detected by nested PCRs

Host (No. tested)	No. (%) positive with:					
	EC9/EC12a SSAP2f/SSAP2r	EE1/EE2 EE3/EE4	ge3a/ge10r ge9f/ge2	EL(569)F/EL(1193)R EL(569)F/EL(1142)R	MAP4AP5/MSP4AP3 msp4f/msp4r	At least one primer
Sheep (*n* = 134)	47 (35.1)	31 (23.1)	9 (6.7)	0 (0)	0 (0)	47 (35.1)
Cattle (*n* = 115)	12 (10.4)	6 (5.2)	3 (2.6)	2 (1.7)	0 (0)	12 (10.4)
Tick (*n* = 114)	16 (14)	5 (4.4)	7 (6.1)	NA^a^	0 (0)	17 (14.9)
Total (*n* = 363)	75 (20.7)	42 (11.6)	19 (5.2)	NA^a^	0 (0)	76 (20.9)

^a^: not applied

**Fig. 1:**
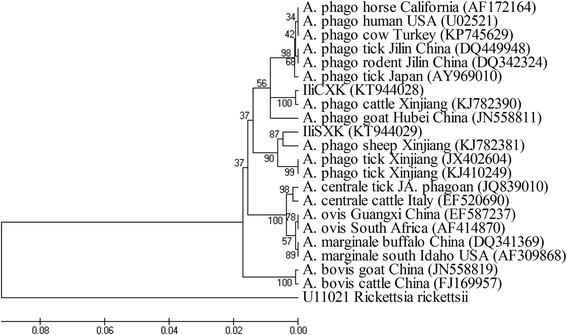
Phylogenetic analysis of *A. phagocytophilum* (*A. phago*) based on 16S rRNA gene partial sequences (599 bp). *Rickettsia rickettsii* is used as an outgroup

The specificity of the assay was controlled by sequencing. Considering the highest sensitivity of the assay with EC primers in this study, 20 samples (four from cattle, seven from sheep, five from *D. marginatus* and four from *H. punctata*) positive for EC primers and the tick sample (*D. marginatus*, sample ID: 2–21, negative for EC but positive for EE) were selected for sequencing. Sequence analyzed by a BLASTn search in GenBank confirmed the presence of *A. phagocytophilum* in those samples. Four sequences (GenBank accession no. KT944028) identified in cattle showed 99.7 % identity to the isolate KS20 (GenBank accession no. KJ782390) from cattle in Kashgar, Xinjiang province. The remaining 16 sequences (GenBank accession no. KT944029) identified in sheep and ticks were identical to each other and showed 99.2 % identity to strain YC38 (GenBank accession no. KJ782381) from sheep in Yecheng, Xinjiang province. Moreover, one sequence (GenBank accession no. KT951192) identified in *D. marginatus* tick (2–21) had 98.9 % identity to the BL102-7 strain (GenBank accession no. KJ410249) derived from *Hyalomma asiaticum* in Xinjiang. Phylogenetic analyses revealed that the *A. phagocytophilum* isolates identified in this study are placed on two separate clades (Fig. 1).

## Discussion

*A. phagocytophilum* is an emerging tick-borne zoonotic agents of public health significance [1]. The disease presents as a clinical syndrome, ranging from asymptomatic to fatal disease [22]. Nonspecific symptoms and signs are manifested in the disease state, and most commonly manifested by fever, chills, headache, and myalgias, which are difficult to differentiate from those of other febrile illness [15, 22]. PCR-based methods are powerful tools and play an important role in the confirmation of *A. phagocytophilum* infection in clinical and environmental specimens. Since the first identification of the HGA agent in 1994, numerous PCR amplification assays and primer sets have been described for detection of *A. phagocytophilum* [1, 23–27]. However, their performance varies [16, 28]. Thus, a choice of PCR methods with appropriate primers that target different DNA segments of *A. phagocytophilum* is crucial for obtaining the best possible results, and this affects the sensitivity and specificity of the diagnostic assays significantly.

*A. phagocytophilum* infection has been reported in humans, wild and domestic animals in China, and the infection rates were variable in different hosts or geographic locations [9, 12, 29, 30]. In this study, the positive rate was significantly higher in sheep than in cattle (Chi2 = 20.781, df = 1, *P* < 0.001), similar result has been described in previous report [29]. Of those tick samples, *A. phagocytophilum* infection was found in *D. marginatus* and *H. punctata* ticks. However, we could not conclude that the role of *D. marginatus* and *H. punctata* as reservoirs or vectors of *A. phagocytophilum* because of the agent detected in this study could be from the hosts, which warrants further investigation.

In the present study, five primer sets, designed to amplify *16S rRNA*, the heat shock gene operon *groESL* and major surface protein gene *msp4*, were used to evaluate the applicability of nested PCR for detection of *A. phagocytophilum* DNA in ruminant and tick specimens. Considerable differences were observed in the performance of these five assays. The results indicated that the nested PCR with EC primer set targeting the 16S rRNA gene is the most sensitive method (Chi2 = 39.944, df = 2, *P* < 0.001). The assay with MSP primer set appeared less useful for detection of *A. phagocytophilum* in ruminants and ticks, and the EL primer set was less effective for detection of tick specimens. The primer sets EE was more sensitive than ge, MSP and EL, but significantly less useful than EC (Chi2 = 11.096, df = 1, *P* < 0.01). However, a previous evaluation of several different published methods for PCR amplification of specific DNA in *A. phagocytophilum*-infected HL-60 cells showed that the ge primer set provided the highest analytical sensitive and specificity, which could detect the equivalent of 0.25 infected cells [16]. Beyond the optimization of the nucleic acid amplification conditions, the PCR amplification efficiency could be influenced by the concentration of DNA templates in this comparative analysis study. Although one tick sample (2–21) was negative for EC but positive for EE, the assay with the EC primer set showed high sensitivity in ruminants and ticks. Sequence analysis of EC–positive samples verified the specificity of the assay. Based on the comparative analysis of those five assays, the nested PCR with EC primer set would provide more reliable results for *A. phagocytophilum* detection in ruminant and tick specimens.

Phylogenetic analysis of the obtained *A. phagocytophilum* 16S rRNA gene sequences in this study showed that they were variable from the sequence from the positive control (Gene accession no. JN558811), excluded possible contamination and indicated the complexity of the genotype of the *A. phagocytophilum* in the field (Fig. 1). The *A. phagocytophilum* strain one (GenBank accession no. KT944028) identified in cattle was most closely related to the isolate detected in cattle from Kashgar (GenBank accession no. KJ782390) (Fig. 1). The strain two (GenBank accession no. KT944029) identified in sheep and ticks were most closely related to the isolate detected in sheep from Yecheng (GenBank accession no. KJ782381) (Fig. 1). These results suggested that *A. phagocytophilum* genotypes are vary between sheep and cattle in sample collection sites.

The results of the present study support the use of nested PCR with primer set EC (EC9/EC12a and SSAP2f/SSAP2r) targeting the *16S rRNA* gene, which was the most sensitive assay for the detection of *A. phagocytophilum* DNA in ruminants and ticks in the region investigated in China. Although this assay was more sensitive than others, it could also miss positive samples for unknown reasons. Given that no test is actually 100 % sensitive or specific, an important consideration is that two or more assays should always be used in parallel to achieve maximum sensitivity for the molecular detection of *A. phagocytophilim*.

There are numerous factors involved in the optimization of PCR assays, and the potential discrepancies make it difficult to ensure the performance of a given assay in different laboratories. *A. phagocytophilum* displayed a high degree of genetic diversity, host tropisms and variation in pathogenicity [31]. Considerable strain variation of *A. phagocytophilum* has been reported in different hosts or geographic locations, and the organism can be genetically divided into several subclusters using *groESL*, *ankA* and *msp4* [17–19]. Recently, four distinct ecological clusters correlate with host species have been established based on *ankA* gene [32], four geographically dispersed ecotypes were identified based on *groESL* gene and showed significantly different host ranges in Europe [33]. Thus, each laboratory should determine the efficacy of those assays under local conditions, and the choice of appropriate assays could yield accurate results for the detection of *A. phagocytophilum* in ticks and animals.

## Conclusions

The performance of five nested PCR assays was accessed by parallel detection of field-collected samples. The nested PCR with primer set EC (EC9/EC12a and SSAP2f/SSAP2r) targeting the *16S rRNA* gene was the most sensitive assay for the detection of *A. phagocytophilum* DNA in ruminants and ticks. *A. phagocytophilum* was detected in 47 (35.1 %) sheep, 12 (10.4 %) cattle, and 17 (14.9 %) ticks collected from Ili Kazakh Autonomous Prefecture in northern Xinjiang, and two *A. phagocytophilum* genotypes were identified. These findings not only provide valuable information for the control of *A. phagocytophilum* infection, but also indicate potential implications for public health.

## Additional file

Additional file 1: Table S1. Nucleotide sequence accession numbers of *A. phagocytophilum*, *A. centrale*, *A. ovis*, *A. marginale*, *A. bovis*, *Anaplasma* sp. and *R. rickettsii* isolates analyzed in this study. (DOCX 35 kb)
