# Supplementary material for: Evaluation of different nested PCRs for detection of Anaplasma phagocytophilum in ruminants and ticks
Source: BMC Vet Res. 2016 Feb 24;12:35. doi: 10.1186/s12917-016-0663-2 (PMC4765105; doi:10.1186/s12917-016-0663-2)
Supplement: Additional file 1: Table S1. — Nucleotide sequence accession numbers of A. phagocytophilum, A. centrale, A. ovis, A. marginale, A. bovis, Anaplasma sp. and R. rickettsii isolates analyzed in this study. (DOCX 35 kb) [file 12917_2016_663_MOESM1_ESM.docx]

Table S1

Nucleotide sequence accession numbers of *A. phagocytophilum*, *A. centrale*, *A. ovis*, *A. marginale*, *A. bovis*, *Anaplasma* sp. and *R. rickettsii* isolates analyzed in this study.

| Strain | Species | Host/source | Geographic origin | Accession no. |
| --- | --- | --- | --- | --- |
| CASOLJ | *Anaplasma phagocytophilum* | Horse | California, USA (1999) | AF172164 |
| Webster | *Anaplasma phagocytophilum* | Human | USA (1994) | U02521 |
| Trbrt45 | *Anaplasma phagocytophilum* | Cow | Turkey (2014) | KP745629 |
| Jilin 5 | *Anaplasma phagocytophilum* | *Dermacentor silvarum* | Jilin, China (2006) | DQ449948 |
| Jilin-1 | *Anaplasma phagocytophilum* | Apodemus agrarius | Jilin, China (2006) | DQ342324 |
| J4-3-1 | *Anaplasma phagocytophilum* | Ixodes ovatus | Japan (2005) | AY969010 |
| KS20 | *Anaplasma phagocytophilum* | Cattle | Xinjiang, China (2012) | KJ782390 |
| YDH6 | *Anaplasma phagocytophilum* | Goat | Hubei, China (2011) | JN558811 |
| YC38 | *Anaplasma phagocytophilum* | Sheep | Xinjiang, China (2012) | KJ782381 |
| Xinjiang051-9 | *Anaplasma* sp. | *Hyalomma asiaticum* | Xinjiang, China (2012) | JX402604 |
| BL102-7 | *Anaplasma* sp. | *Hyalomma asiaticum* | Xinjiang, China (2011) | KJ410249 |
| C4B | *Anaplasma centrale* | *Boophilus microplus* | Cebu, Philippines (2011) | JQ839010 |
| 16 | *Anaplasma centrale* | Cattle | Italy (2007) | EF520690 |
| CGX | *Anaplasma ovis* | NA | Guangxi, China (2007) | EF587237 |
| OVI | *Anaplasma ovis* | NA | South Africa (2001) | AF414870 |
| NA | *Anaplasma marginale* | Buffalo | Southern China (2005) | DQ341369 |
| South Idaho | *Anaplasma marginale* | NA | Idaho, USA (2000) | AF309868 |
| B7 | *Anaplasma bovis* | Goat | Zhejiang, China (2011) | JN558819 |
| Zhongxian | *Anaplasma bovis* | Cattle | Chongqing, China (2008) | FJ169957 |
| Sawtooth | *Rickettsia rickettsii* | *Dermacentor andersonii* | NA | U11021 |
| IliCXK | *Anaplasma phagocytophilum* | Cattle | In this study | KT944028 |
| IliSXK | *Anaplasma phagocytophilum* | Sheep | In this study | KT944029 |

NA: Not available.
